# Quantitative proteomic analysis of formalin–fixed, paraffin–embedded clear cell renal cell carcinoma tissue using stable isotopic dimethylation of primary amines

**DOI:** 10.1186/s12864-015-1768-x

**Published:** 2015-07-29

**Authors:** J. Weißer, Z. W. Lai, P. Bronsert, M. Kuehs, V. Drendel, S. Timme, S. Kuesters, C. A. Jilg, U. F. Wellner, S. Lassmann, M. Werner, M. L. Biniossek, O. Schilling

**Affiliations:** Institute of Molecular Medicine and Cell Research, University of Freiburg, Freiburg, Germany; Present address: CeMM Research Center for Molecular Medicine of the Austrian Academy of Sciences, A-1090 Vienna, Austria; Department of Pathology, University Medical Center Freiburg, Freiburg, Germany; Clinic for General and Visceral Surgery, University Medical Center Freiburg, Freiburg, Germany; Present address: Clinic for Surgery, University Clinic of Schleswig-Holstein Campus Lübeck, Lübeck, Germany; BIOSS Centre for Biological Signaling Studies, University of Freiburg, D-79104 Freiburg, Germany; Comprehensive Cancer Center Freiburg, Freiburg, Germany; Urologische Klinik und Zentrale Klinische Forschung, Klinikum der Universität Freiburg, Freiburg, 79106 Germany; German Cancer Consortium (DKTK) and German Cancer Research Center (DKFZ), Heidelberg, Germany

**Keywords:** Dimethylation, Formalin-fixation, Paraffin-embedment clear cell renal cell carcinoma

## Abstract

**Background:**

Formalin-fixed, paraffin-embedded (FFPE) tissues represent the most abundant resource of archived human specimens in pathology. Such tissue specimens are emerging as a highly valuable resource for translational proteomic studies. In quantitative proteomic analysis, reductive di-methylation of primary amines using stable isotopic formaldehyde variants is increasingly used due to its robustness and cost-effectiveness.

**Results:**

In the present study we show for the first time that isotopic amine dimethylation can be used in a straightforward manner for the quantitative proteomic analysis of FFPE specimens without interference from formalin employed in the FFPE process. Isotopic amine dimethylation of FFPE specimens showed equal labeling efficiency as for cryopreserved specimens. For both FFPE and cryopreserved specimens, differential labeling of identical samples yielded highly similar ratio distributions within the expected range for dimethyl labeling. In an initial application, we profiled proteome changes in clear cell renal cell carcinoma (ccRCC) FFPE tissue specimens compared to adjacent non–malignant renal tissue. Our findings highlight increased levels of glyocolytic enzymes, annexins as well as ribosomal and proteasomal proteins.

**Conclusion:**

Our study establishes isotopic amine dimethylation as a versatile tool for quantitative proteomic analysis of FFPE specimens and underlines proteome alterations in ccRCC.

**Electronic supplementary material:**

The online version of this article (doi:10.1186/s12864-015-1768-x) contains supplementary material, which is available to authorized users.

## Background

Formalin − fixation, paraffin − embedding (FFPE) is routinely used to preserve human tissue samples for routine pathological diagnostics. Comprehensive archives of FFPE specimens have been established in most large Institutes of Pathology, harboring tissue specimens from most human diseases, including comparably rare medical conditions. As these specimens are carefully annotated regarding diagnosis, treatment and patient outcome, they are a unique research resource for proteomic analysis aiming to identify key proteins in disease progression and treatment response.

In recent years considerable advances have been made regarding the proteomic analysis of FFPE specimens. Upon formalin fixation, extensive protein cross-linking occurs, largely involving the ε–amino group of lysine residues. These covalent cross-links are reversed by sample heating in the presence of a strong detergent (*e.g.* sodium dodecyl sulfate, SDS) or a chaotropic salt [1–3]. FFPE samples that were > 5 years old have been successfully analyzed by liquid chromatography (LC) − tandem mass spectrometry (MS/MS) [4–7]. There have been considerable improvements with regard to proteome coverage [2, 8, 9] and the ability to analyse post–translational modifications such as glycosylation or phosphorylation [8].

Quantitative proteomic analysis of FFPE specimens has been performed using two–dimensional differential gel electrophoresis [10] or label–free chromatographic approaches [2, 5, 11]. Chemical isotope tagging of FFPE samples has been performed using iTRAQ labeling [12–15].

Reductive dimethylation of primary amines is a widely used strategy for relative quantification of peptides and proteins by LC-MS/MS [16]. Distinguishing features of dimethyl labeling are its robustness and cost–effectiveness together with the ability of binary and triplex approaches [16]. It has recently been shown that quantification with dimethyl labeling is as accurate as metabolic labeling strategies [17], which have been referred to as the gold standard in quantitative proteomics [18].

In this study we establish the usage of dimethyl labeling for the quantitative proteomic investigation of FFPE specimens. Using corresponding cryopreserved tissue specimens as controls, we show that formalin fixation and paraffin embedding does not interfere with the formaldehyde–based dimethyl labeling. In an exemplary proof–of–concept application, we used dimethyl labeling to profile proteome changes in FFPE tissue specimens of clear cell renal cell carcinoma (ccRCC), comparing ccRCC to adjacent non–malignant renal cells. We found elevated levels of glycolytic enzymes that are in line with previous studies.

Our findings add robust and cost–effective dimethyl labeling to the toolbox for quantitative proteomic analysis of FFPE specimens as well as providing further proteomic insight into ccRCC pathology.

## Results and discussion

### Overview

The present study aims to evaluate isotopic dimethyl labeling for the quantitative proteomic analysis of FFPE tissue specimens. This stable isotope tagging method is then applied for quantitative proteome profiling of ccRCC tissue in comparison to adjacent non-malignant tissue.

### Labeling efficiency

**Fig. 1:**
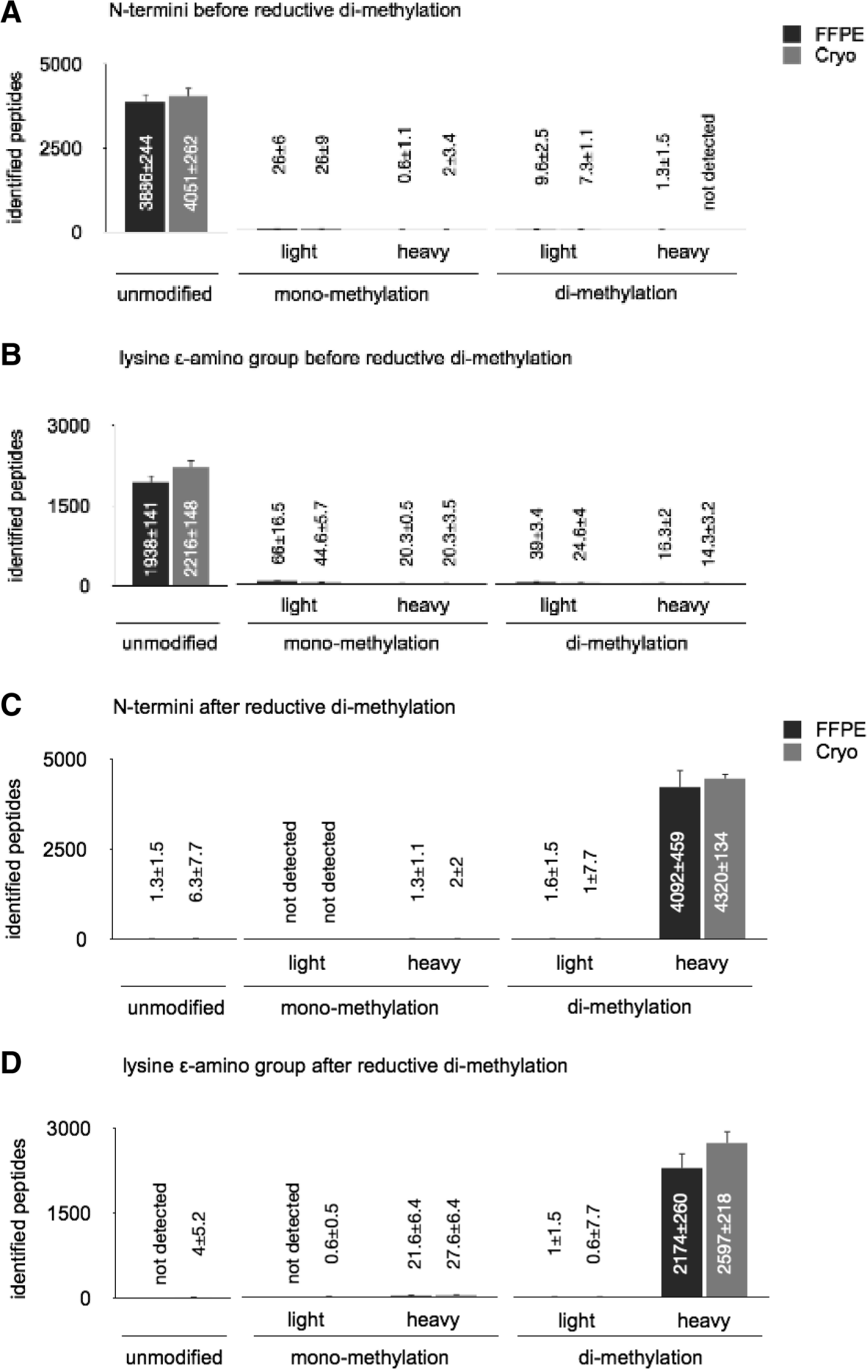
Modifications present at N-termini and lysine side chains before and after reductive dimethylation with either light (^12^COH_2_) or heavy (^13^COD_2_) formaldehyde. Data represents average values ± standard deviation; based on the analysis of three proteome samples from solid tumors. Identification numbers (average values ± standard deviation) are stated. **a**) modifications of peptide N-termini before di-methylation; **b**) modifications of lysine side-chains before dimethylation; **c**) modifications of peptide N-termini after di-methylation; **d**) modifications of lysine side-chains after di-methylation

We initially profiled FFPE–derived protein samples for the occurrence of dimethylated primary amines (α-amines of peptide N–termini and ε–amines of lysine side chains). For FFPE samples and cryopreserved non-labeled control protein samples, we did not detect relevant levels of mono– or dimethylated peptides N-termini (Fig. 1a) or lysine side chains (Fig. 1b). Several reports indicate that FFPE protein samples are susceptible to a +12 Da addition at N-termini as well as lysine, tryptophan, and tyrosine residues [19–21]. Using the non-dimethylated protein samples, we probed for occurrence of these modifications. For both FFPE and cryopreserved samples, less than 5 % of the identified peptides showed these modifications (not shown).

Next, we assessed whether FFPE–derived protein samples are amenable for dimethyl labeling on the peptide level after trypsination. To discriminate between the chemically introduced label and arbitrarily introduced modifications (*e.g.* formalin traces in the FFPE samples), we used heavy formaldehyde (^13^COD_2_). For both FFPE samples and cryopreserved control samples, dimethylation works robustly as > 95 % of all identified peptides were present in the heavy dimethylated form with regard to their N–termini (Fig. 1c) as well as lysine side chains (Fig. 1d). We did not detect relevant levels of unlabeled, monomethylated or light-dimethylated peptides.

Protein samples derived from both FFPE or cryopreserved control tissue specimens yield comparable peptide identification numbers, which are in the range of recent reports of proteomic analysis of FFPE tissues [6, 7, 11, 22]. Together, these results highlight that FFPE specimens are amenable to dimethyl labeling without interference from the initial formalin fixation.

### Quantitation accuracy

**Fig. 2:**
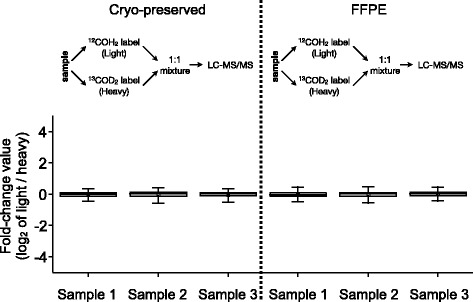
Quantitative proteomic comparison of identical samples that were split, labeled by reductive dimethylation with either light (^12^COH_2_) or heavy (^13^COD_2_) formaldehyde, and mixed 1:1. Box-and-whisker plots denote the 25–75 percentile and the 5 – 95 percentile, respectively. Data represents average values ± standard deviation; based on the analysis of three proteome samples from solid tumors. Protein and peptide identification numbers are 572 proteins/4528 unique peptides for cryo-sample 1; 637 proteins/4627 unique peptides for cryo-sample 2; 605 proteins/4763 unique peptides for cryo-sample 3; 647 proteins/4977 unique peptide for FFPE-sample 1; 564 proteins/4113 unique peptides for FFPE-sample 2; 601 proteins/4367 unique peptides for FFPE sample 3

To investigate whether dimethyl labeling yields accurate quantification of FFPE –derived protein samples, we halved FFPE samples after trypsination, labeled the different aliquots with either light (^12^COH_2_) or heavy (^13^COD_2_) formaldehyde, and mixed the aliquots at a 1:1 ratio, followed by LC–MS/MS analysis (Fig. 2). The same procedure was conducted with cryopreserved control samples (Fig. 2). The analysis was performed in triplicate, yielding consistently between 560 and 540 protein identifications. The Fc-values (log_2_ of the L:H ratio) display a narrow distribution, with almost identical standard deviations, ranging from 0.34 – 0.41 for the cryopreserved samples and from 0.33 – 0.41 for the FFPE samples. A recent benchmarking study of dimethyl labeling showed a standard deviation of 0.34 for a 1:0.5 mix of an identical sample that was differentially labeled using dimethylation [17]. Our results are in very good agreement with the previously reported outcome. Recently, Wakabayashi *et al.* have also shown applicability of reductive dimethylation to FFPE-extracted phosphopeptides [23].

In summary, FFPE tissue specimens are amenable to protein extraction and subsequent relative quantitation by isotopic dimethyl labeling in LC-MS/MS analysis.

### Application to quantitative proteome profiling of clear cell renal cell carcinoma

Clear cell renal cell carcinoma (ccRCC) is among the ten most common human malignancies and accounts for more than 90 % of renal neoplasms. As it is clinically occult in many patients, diagnosis often occurs only at a later stage of disease and approximately one third of patients presents with metastatic disease upon diagnosis. Response rates to treatment are generally low for metastatic ccRCC, leading to a median survival of less than one year [24, 25].

As a first application of isotopic dimethyl labeling for relative protein quantitation, we focused on ccRCC. Four cases of ccRCC were analyzed, in which tumor and adjacent normal tissue was represented in the same FFPE tissue blocks. In accordance with the workflow established in this study, we used “light” and “heavy” dimethylation with isotopic formaldehyde for quantitative proteomic comparison of the protein samples derived from the malignant and non–malignant FFPE specimen areas. Protein samples of each case were analyzed separately. To increase proteome coverage, we employed SCX prefractionation.

**Fig. 3:**
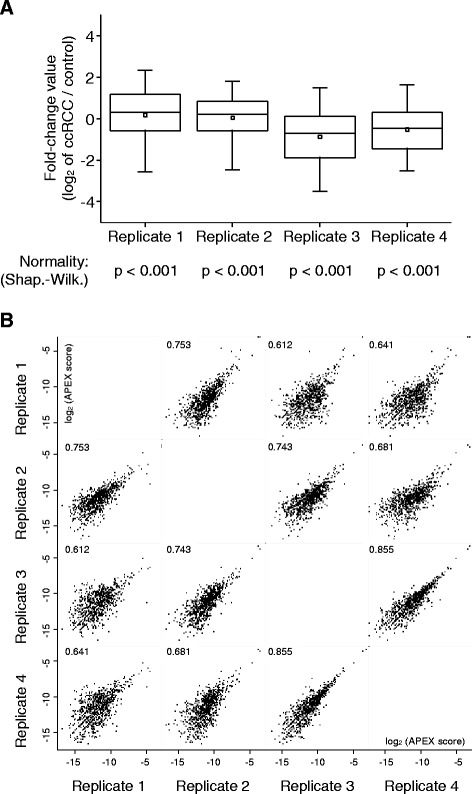
Quantitative proteomic comparison of FFPE derived ccRCC tumor tissue with FFPE derived, adjacent non-malignant tissue. Stable isotope labeling was achieved by reductive dimethylation with either light (^12^COH_2_) or heavy (^13^COD_2_) formaldehyde. **a** Box-and-whisker plots denoting the 25–75 percentile and the 5 – 95 percentiles, respectively, of the four replicates (**b**) Correlation of APEX scores [26, 27], showing the Pearson correlation. Protein and peptide identification numbers are 1518 proteins/6619 unique peptides for replicate 1; 2490 proteins/13501 unique peptides for replicate 2; 1590 proteins/12811 peptides for replicate 3; 1352 proteins/9207 peptides for replicate 4

The Fc-values of each replicate experiment followed a near-normal distribution (Shapiro–Wilk test, Fig. 3a). Peptides with unlabeled N-termini or lysine side-chains constitute less than 3 % of all identified peptides (data not shown). As expected, the Fc-values are broadly distributed in each replicate (Fig. 3a), indicating substantial proteome differences between ccRCC tissue and adjacent, non-malignant tissue. We used the APEX method to calculate protein abundances [26, 27]. The resulting APEX scores displayed good correlation between the different replicates (Fig. 3b).

A total of 2938 non-redundant proteins were identified, 1307 of these were found in at least three replicates. As previously described, we employed the following criteria to distinguish significantly affected proteins: (A) identification in at least three replicate experiments, (B) protein abundance differences resulted in a p-value < 0.05 (2-tailed Student’s *t* test with Benjamini-Hochberg correction for multiple testing at an FDR < 0.05), (C) protein abundance increased or decreased in with an average Fc-value > 0.58 or < −0.58 (equivalent to an abundance change > 50 %). With these criteria, 112 proteins were found to be increased in ccRCC tissue (Additional file 1: Table S1) whereas 77 proteins were found to be decreased in ccRCC tissue in comparison to adjacent non-malignant tissue (Additional file 1: Table S2).

**Fig. 4:**
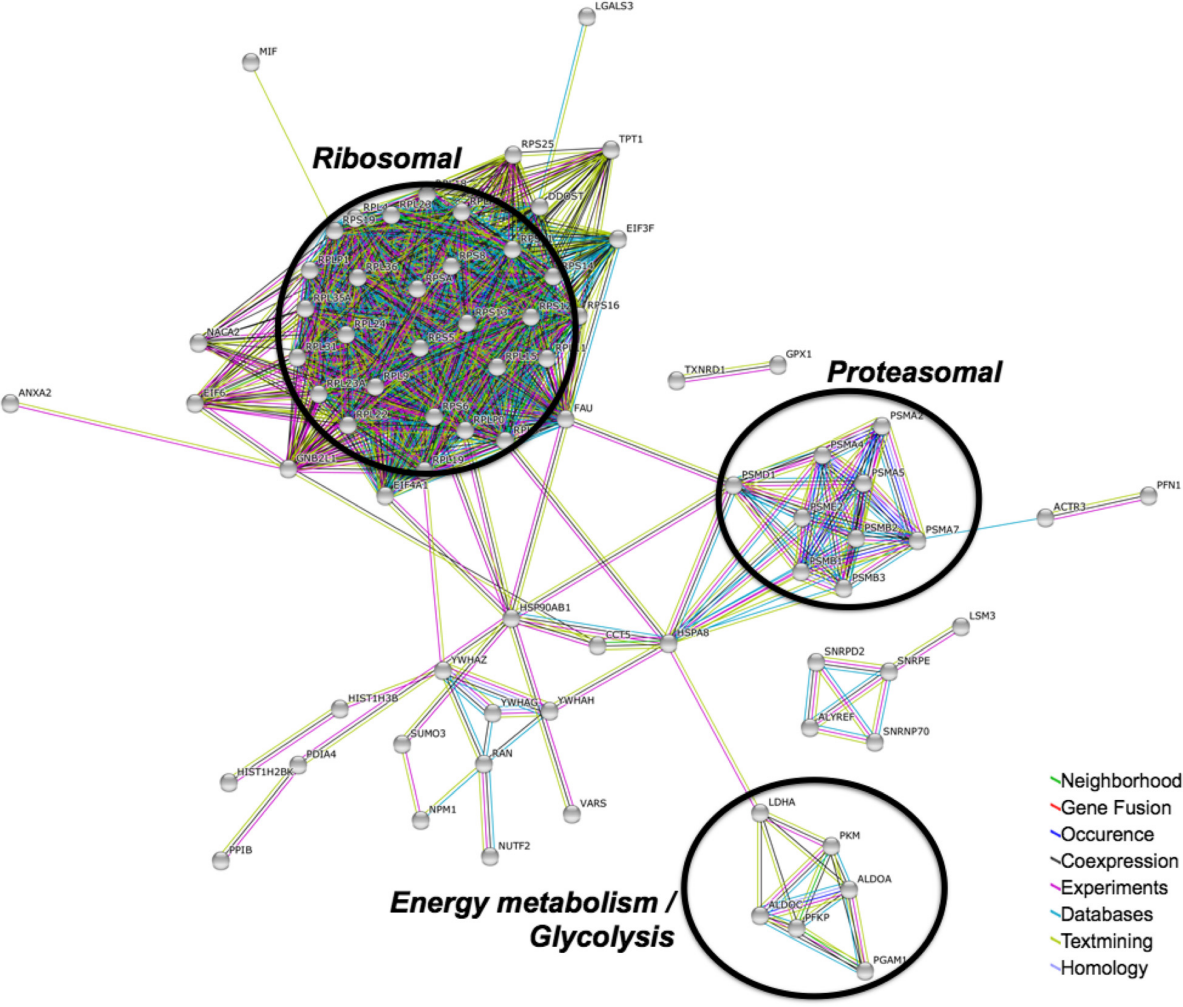
STRING protein functional association network [30] of proteins that were found to be significantly upregulated in ccRCC compared to adjacent non-malignant tissue (*p*-value < 0.05, 2-tailed Student *t*-test, at least 50 % increased abundance). STRING was employed using “high confidence”. Disconnected nodes are not shown. Connections are shown using standard STRING coloring scheme as highlighted in the legend

The online Search Tool for the Retrieval of Interacting Genes (STRING) was used to display connections between proteins with either elevated or decreased levels [28]. STRING visualized several functional clusters for proteins that were found to be elevated in ccRCC (Fig. 4), including ribosomal and proteasomal proteins as well as proteins involved in glycolysis and energy metabolism.

Our finding of increased levels of proteins involved in glycolysis in ccRCC, compared to corresponding non-malignant kidney tissue is in line with further reports, including proteomic, metabolomic, and functionally genomic approaches; all of which point towards increased aerobic glycolysis (“Warburg effect”) in ccRCC [25, 29–31].

Although not highlighted by a STRING cluster, we noticed elevated levels of annexins A2 and A4. This is corroborated by further proteomic studies on ccRCC [25, 29, 31]. For annexins II and IV, tumor promoting roles in ccRCC have been suggested, based on *in vitro* findings indicating that annexin II and IV, respectively, sustain tumor cell migration [32, 33].

Our proteomic analysis of ccRCC further suggests elevated levels of ribosomal and proteasomal proteins, putatively indicating a generally increased protein turnover in ccRCC as compared to non-malignant kidney tissue. Previous proteomic analyses of ccRCC have not highlighted a ribosomal/proteasomal fingerprint. There are however several reports that link proteasomal function to ccRCC. For example, exome sequencing has previously shown that components of the ubiquitin system are frequently mutated in ccRCC [34]. Inactivation of the nuclear deubiquitinating enzyme BAP1 is also a frequent event in ccRCC [35]. Moreover, increased serum levels of the 20 S proteasome have been found in ccRCC patients [36]. We identified 28 proteasomal proteins. All of these were increased in ccRCC with the exceptions of proteasome subunit β type-5 and type-7. 11 proteasomal proteins met our criteria for significantly increased abundance. Likewise, we identified 72 ribosomal or ribosome-associated proteins excluding mitochondrial ribosomal proteins. All of these were increased in ccRCC with the exceptions of ribosome-binding protein 1 and 60S ribosomal protein L34. 30 proteasomal proteins met our criteria for significantly increased abundance.

In summary, application of isotopic dimethyl tagging allowed for the quantitative profiling of ccRCC tissue. Our analysis corroborated previously established proteome motifs of ccRCC, *i.e.* aerobic glycolysis, as well as pointing to newly discovered proteome alterations, *i.e.* increased levels of both ribosomal and proteasomal proteins.

Our exemplary application focuses on a pairwise comparison of different samples. Dimethyl labeling is typically restricted to pairwise or triple comparisons. We consider this setting to be its typical application. This limitation can be overcome by using a differentially dimethylated standard sample, analogous to the Super-SILAC strategy [37]. By comparing multiple samples against this standard, a larger number of samples can be probed.

## Conclusion

Our results show that dimethyl labeling is applicable for the quantitative proteomic analysis of FFPE tissue specimens without interference from the formalin fixation process. Quantitation accuracy is comparable to cryopreserved tissue specimens. An initial application of dimethyl labeling to FFPE specimens portrayed differences in the proteome composition of ccRCC compared to adjacent non–malignant tissue. Dimethyl labeling with isotopic formaldehyde is a robust and cost–effective labeling strategy for quantitative proteomics. The present work adds dimethyl labeling to the toolbox for quantitative proteome analysis of FFPE specimens.

## Methods

### Samples

As proof-of-principle tissue specimens for labeling experiments, samples were derived from large solid tumors. From each tissue specimen, one piece was immediately fixed with formalin and embedded in paraffin, the other was immediately snap-frozen in liquid nitrogen and stored at −80 °C. As a clinical application, four FFPE tissue specimens of clear cell renal cell carcinoma (ccRCC) and adjacent non-malignant kidney tissue were chosen. Routine protocols of the Institute of Surgical Pathology were used for all proof-of-principle and ccRCC samples.

For all tissue specimens, diagnosis was confirmed by experienced pathologists. All tissue specimens were processed within 20 min after surgical removal. After processing, samples were immediately anonymized. No tumor showed macroscopical or microscopical signs of necrosis. Routine diagnostics was not affected. The study was approved by the Ethics Committee of the Medical University Freiburg, (311/12_130523, “Feingewebliche, immunhistochemische und molekularpathologische Untersuchungen von benignem und malignem Gewebe urogenitaler Tumore sowie korrespondierender Metastasen aus Formalin-fixiertem, Paraffin-eingebettetem und Frischgewebe.”/“Histological, immunohistochemical, and molecular-pathological investigations of benign and malignant tissues of urogenital tumours and corresponding metastases from formalin-fixed, paraffin-embedded tissues”). Before study inclusion, all patient data were anonymized. Informed consent was obtained from all participants.

### Sample preparation

10 μm slides were cut from FFPE specimens and were deparaffinized with xylene, rehydrated in a decreasingly graded ethanol series and transferred into microreaction tubes. Cryopreserved specimens were carefully crushed with a scalpel. All tissue specimens were incubated in 100 mM 4-(2-hydroxyethyl)-1-piperazineethanesulfonic acid (HEPES) pH 7.5, 4 % (w/v) sodium dodecyl sulfate (SDS), 50 mM dithiothreitol (DTT) for 1 h at 95 °C with gentle rotation. Typically, 150 μl buffer was used for approximately 10 FFPE slices of 10 μm thickness. Proteins were precipitated by addition of 9 volumes of acetone and 1 volume of methanol and incubated at −80 °C for 2 h. After washing with methanol, the proteins were resuspended in 100 mM NaOH aided by sonication at 4 °C and the solution was brought to pH 8.0 with 200 mM HEPES free acid. Protein concentrations were determined using BCA (Pierce) and Bradford (Bio-Rad) assays. Typical protein yield after acetone precipitation was in the range of 1.0 mg from 10 FFPE slices of 10 μm thickness. Proteins (up to 500 μg) were trypsinized using sequencing grade trypsin (Worthington, 1:100, 18 h at 37 °C). Cysteine residues were reduced and alkylated. If applicable, primary amines were reductively di-methylated in solution (200 mM HEPES, pH 8.0) by addition of 40 mM formaldehyde (^12^COH_2_,light‘(Sigma) or ^13^COD_2_,heavy’ (Cambridge Isotopes)) and 40 mM sodium cyanoborohydride (pH 8.0, 37 °C 18 h, (Sigma)). Excess reagents were quenched with 20 mM glycine (20 min, 22 °C). If applicable, equal amounts of amounts of heavy and light labelled samples were mixed. Before mass spectrometric analysis, all samples were desalted using self packed C18 Stage-tips [38]. ccRCC proteome comparison samples were pre-fractionated using strong cation exchange (SCX) chromatography as described previously [39, 40].

### LC-MS/MS analysis

Analysis was performed on an Orbitrap XL (Thermo Scientific) mass spectrometer that was coupled to an Ultimate3000 micro pump (Thermo Scientific). Buffer A was 0.5 % acetic acid, buffer B 0.5 % acetic acid in 80 % acetonitrile (HPLC grade). Liquid phases were applied at a flow rate of 300 nl/min with an increasing gradient of organic solvent for peptide separation. Reprosil-Pur 120 ODS-3 (Dr. Maisch) was used to pack column tips of 75 μm inner diameter and 11 cm length. The MS was operated in data dependent mode and each MS scan was followed by a maximum of five MS/MS scans.

### LC-MS/MS data analysis

LC-MS/MS data was obtained in raw format and converted to the mzXML [41] format, using msconvert [42] with centroiding of MS1 and MS2 data, and deisotoping of MS2 data. For spectrum to sequence assignment X! Tandem (version 2013.09.01) [43] was used. The proteome database consisted of human reviewed canonical uniprot sequences (without isoforms, 20,240 protein entries) downloaded from UniProt on November 26th, 2013, appended with an equal number of shuffled decoy entries derived from the original human protein sequences (DB toolkit, [44]). Two different searches were conducted for light and heavy labeled peptides. X! Tandem parameters included: pre-cursor mass error of 10 ppm, fragment ion mass tolerance of 0.3 Da, tryptic cleavage specificity with up to three missed cleavages for probing labeling efficiency and up to one missed cleavage when applying the labeling technique to tumor samples. Residue modifications: cysteine carboxyamidomethylation (+57.02 Da), lysine and N-terminal dimethylation (light formaldehyde 28.03 Da; heavy formaldehyde 34.06 Da); no variable modifications. X! Tandem results were further validated by PeptideProphet [45] at a confidence level of > 95 %. Peptides were assembled to proteins using ProteinProphet [46] with a false discovery rate (FDR) < 1.0 %. For relative peptide and protein quantification XPRESS [47] was used. Mass tolerance for quantification was 0.02 Da. XPRESS data was log_2_-transformed yielding fold change (Fc)–values. For the ccRCC replicate analyses, protein abundance was considered to be significantly altered if the following conditions were met: (A) the protein was identified in at least three replicate experiments, (B) protein abundance was significantly increased or decreased (p-value < 0.05, based on 2-tailed Student’s *t* test with Benjamini-Hochberg correction for multiple testing at an FDR < 0.05; the Perseus framework was used for statistical analysis [48]), (C) protein abundance increased or decreased with an average Fc-value > 0.58 or < −0.58.

### Supporting data

The LC-MS/MS data underlying this study were uploaded to the PeptideAtlas database and can be retrieved at http://www.peptideatlas.org/PASS/PASS00702.

## Additional file

Additional file 1: Table S1. Proteins with significantly increased abundance in ccRCC compared to adjacent non-malignant tissue (p-value < 0.05, 2-tailed Student *t*-test, see main text for further details). Empty cells indicate that a protein was not identified in a replicate. **Table S2.** Proteins with significantly increased abundance in ccRCC compared to adjacent non-malignant tissue (*p*-value < 0.05, 2-tailed Student *t*-test, see main text for further details). Empty cells indicate that a protein was not identified in a replicate.

## Abbreviations

ccRCC: Clear cell renal cell carcinoma
DTT: Dithiothreitol
FDR: False discovery rate
FFPE: Formalin-fixed, paraffin-embedded
HEPES: 4-(2-hydroxyethyl)-1-piperazineethanesulfonic acid
LC: Liquid chromatography
MS/MS: Tandem mass spectrometry
SCX: Strong cation exchange
SDS: Sodium dodecyl sulfate

## References

[1] Shi S-R, Taylor CR, Fowler CB, Mason JT (2013). Complete solubilization of formalin-fixed, paraffin-embedded tissue may improve proteomic studies. Proteomics Clin Appl.

[2] Wiśniewski JR, Ostasiewicz P, Mann M (2011). High recovery FASP applied to the proteomic analysis of microdissected formalin fixed paraffin embedded cancer tissues retrieves known colon cancer markers. J Proteome Res.

[3] Jiang X, Jiang X, Feng S, Tian R, Ye M, Zou H (2007). Development of efficient protein extraction methods for shotgun proteome analysis of formalin-fixed tissues. J Proteome Res.

[4] Balgley BM, Guo T, Zhao K, Fang X, Tavassoli FA, Lee CS (2009). Evaluation of archival time on shotgun proteomics of formalin-fixed and paraffin-embedded tissues. J Proteome Res.

[5] Craven RA, Cairns DA, Zougman A, Harnden P, Selby PJ, Banks RE (2013). Proteomic analysis of formalin-fixed paraffin-embedded renal tissue samples by label-free MS: assessment of overall technical variability and the impact of block age. Proteomics Clin Appl.

[6] Sprung RW, Brock JWC, Tanksley JP, Li M, Washington MK, Slebos RJC (2009). Equivalence of protein inventories obtained from formalin-fixed paraffin-embedded and frozen tissue in multidimensional liquid chromatography-tandem mass spectrometry shotgun proteomic analysis. Mol Cell Proteomics.

[7] Fu Z, Yan K, Rosenberg A, Jin Z, Crain B, Athas G (2013). Improved protein extraction and protein identification from archival formalin-fixed paraffin-embedded human aortas. Proteomics Clin Appl.

[8] Ostasiewicz P, Zielinska DF, Mann M, Wiśniewski JR (2010). Proteome, phosphoproteome, and N-glycoproteome are quantitatively preserved in formalin-fixed paraffin-embedded tissue and analyzable by high-resolution mass spectrometry. J Proteome Res.

[9] Wiśniewski JR, Duś K, Mann M (2013). Proteomic workflow for analysis of archival formalin-fixed and paraffin-embedded clinical samples to a depth of 10 000 proteins. Proteomics Clin Appl.

[10] Tanca A, Pisanu S, Biosa G, Pagnozzi D, Antuofermo E, Burrai GP (2013). Application of 2D-DIGE to formalin-fixed diseased tissue samples from hospital repositories: results from four case studies. Proteomics Clin Appl.

[11] Piersma SR, Warmoes MO, de Wit M, de Reus I, Knol JC, Jiménez CR (2013). Whole gel processing procedure for GeLC-MS/MS based proteomics. Proteome Sci.

[12] Jain MR, Liu T, Hu J, Darfler M, Fitzhugh V, Rinaggio J (2008). Quantitative proteomic analysis of formalin fixed paraffin embedded oral HPV lesions from HIV patients. Open Proteomics J.

[13] Xiao Z, Li G, Chen Y, Li M, Peng F, Li C (2010). Quantitative proteomic analysis of formalin-fixed and paraffin-embedded nasopharyngeal carcinoma using iTRAQ labeling, two-dimensional liquid chromatography, and tandem mass spectrometry. J Histochem Cytochem.

[14] Jain MR, Li Q, Liu T, Rinaggio J, Ketkar A, Tournier V (2012). Proteomic identification of immunoproteasome accumulation in formalin-fixed rodent spinal cords with experimental autoimmune encephalomyelitis. J Proteome Res.

[15] Nakatani S, Wei M, Ishimura E, Kakehashi A, Mori K, Nishizawa Y (2012). Proteome analysis of laser microdissected glomeruli from formalin-fixed paraffin-embedded kidneys of autopsies of diabetic patients: nephronectin is associated with the development of diabetic glomerulosclerosis. Nephrol Dial Transplant.

[16] Boersema PJ, Raijmakers R, Lemeer S, Mohammed S, Heck AJR (2009). Multiplex peptide stable isotope dimethyl labeling for quantitative proteomics. Nat Protoc.

[17] Altelaar AFM, Frese CK, Preisinger C, Hennrich ML, Schram AW, Timmers HTM (2013). Benchmarking stable isotope labeling based quantitative proteomics. J Proteome.

[18] Ong S-E, Mann M (2005). Mass spectrometry-based proteomics turns quantitative. Nat Chem Biol.

[19] Metz B, Kersten GF, Baart GJ, de Jong A, Meiring H, ten Hove J (2006). Identification of formaldehyde-induced modifications in proteins: reactions with insulin. Bioconjug Chem.

[20] Tanca A, Abbondio M, Pisanu S, Pagnozzi D, Uzzau S, Addis MF (2014). Critical comparison of sample preparation strategies for shotgun proteomic analysis of formalin-fixed, paraffin-embedded samples: insights from liver tissue. Clin Proteomics.

[21] Toews J, Rogalski JC, Clark TJ, Kast J (2008). Mass spectrometric identification of formaldehyde-induced peptide modifications under in vivo protein cross-linking conditions. Anal Chim Acta.

[22] Gámez-Pozo A, Ferrer NI, Ciruelos E, López-Vacas R, Martínez FG, Espinosa E (2013). Shotgun proteomics of archival triple-negative breast cancer samples. Proteomics Clin Appl.

[23] Wakabayashi M, Yoshihara H, Masuda T, Tsukahara M, Sugiyama N, Ishihama Y (2014). Phosphoproteome analysis of formalin-fixed and paraffin-embedded tissue sections mounted on microscope slides. J Proteome Res.

[24] Gupta K, Miller JD, Li JZ, Russell MW, Charbonneau C (2008). Epidemiologic and socioeconomic burden of metastatic renal cell carcinoma (mRCC): a literature review. Cancer Treat Rev.

[25] Perroud B, Ishimaru T, Borowsky AD, Weiss RH (2009). Grade-dependent proteomics characterization of kidney cancer. Mol Cell Proteomics.

[26] Braisted JC, Kuntumalla S, Vogel C, Marcotte EM, Rodrigues AR, Wang R (2008). The APEX Quantitative Proteomics Tool: generating protein quantitation estimates from LC-MS/MS proteomics results. BMC Bioinformatics.

[27] Lu P, Vogel C, Wang R, Yao X, Marcotte EM (2007). Absolute protein expression profiling estimates the relative contributions of transcriptional and translational regulation. Nat Biotechnol.

[28] Szklarczyk D, Franceschini A, Kuhn M, Simonovic M, Roth A, Minguez P (2011). The STRING database in 2011: functional interaction networks of proteins, globally integrated and scored. Nucleic Acids Res.

[29] White NM, Masui O, Desouza LV, Krakovska O, Metias S, Romaschin AD (2014). Quantitative proteomic analysis reveals potential diagnostic markers and pathways involved in pathogenesis of renal cell carcinoma. Oncotarget.

[30] Cancer Genome Atlas Research N (2013). Comprehensive molecular characterization of clear cell renal cell carcinoma. Nature.

[31] Perroud B, Lee J, Valkova N, Dhirapong A, Lin PY, Fiehn O (2006). Pathway analysis of kidney cancer using proteomics and metabolic profiling. Mol Cancer.

[32] Zimmermann U, Balabanov S, Giebel J, Teller S, Junker H, Schmoll D (2004). Increased expression and altered location of annexin IV in renal clear cell carcinoma: a possible role in tumour dissemination. Cancer Lett.

[33] Yang SF, Hsu HL, Chao TK, Hsiao CJ, Lin YF, Cheng CW (2015). Annexin A2 in renal cell carcinoma: expression, function, and prognostic significance. Urol Oncol.

[34] Guo G, Gui Y, Gao S, Tang A, Hu X, Huang Y (2012). Frequent mutations of genes encoding ubiquitin-mediated proteolysis pathway components in clear cell renal cell carcinoma. Nat Genet.

[35] Pena-Llopis S, Vega-Rubin-de-Celis S, Liao A, Leng N, Pavia-Jimenez A, Wang S (2012). BAP1 loss defines a new class of renal cell carcinoma. Nat Genet.

[36] de Martino M, Hoetzenecker K, Ankersmit HJ, Roth GA, Haitel A, Waldert M (2012). Serum 20S proteasome is elevated in patients with renal cell carcinoma and associated with poor prognosis. Br J Cancer.

[37] Geiger T, Cox J, Ostasiewicz P, Wisniewski JR, Mann M (2010). Super-SILAC mix for quantitative proteomics of human tumor tissue. Nat Methods.

[38] Rappsilber J, Ishihama Y, Mann M (2003). Stop and go extraction tips for matrix-assisted laser desorption/ionization, nanoelectrospray, and LC/MS sample pretreatment in proteomics. Anal Chem.

[39] Tholen S, Biniossek ML, Gansz M, Gomez-Auli A, Bengsch F, Noel A (2013). Deletion of cysteine cathepsins B or L yields differential impacts on murine skin proteome and degradome. Mol Cell Proteomics.

[40] Tholen S, Biniossek ML, Gessler AL, Muller S, Weisser J, Kizhakkedathu JN (2011). Contribution of cathepsin L to secretome composition and cleavage pattern of mouse embryonic fibroblasts. Biol Chem.

[41] Pedrioli PG, Eng JK, Hubley R, Vogelzang M, Deutsch EW, Raught B (2004). A common open representation of mass spectrometry data and its application to proteomics research. Nat Biotechnol.

[42] Kessner D, Chambers M, Burke R, Agus D, Mallick P (2008). ProteoWizard: open source software for rapid proteomics tools development. Bioinformatics.

[43] Craig R, Beavis RC (2004). TANDEM: matching proteins with tandem mass spectra. Bioinformatics.

[44] Martens L, Vandekerckhove J, Gevaert K (2005). DBToolkit: processing protein databases for peptide-centric proteomics. Bioinformatics.

[45] Keller A, Nesvizhskii AI, Kolker E, Aebersold R (2002). Empirical statistical model to estimate the accuracy of peptide identifications made by MS/MS and database search. Anal Chem.

[46] Nesvizhskii AI, Keller A, Kolker E, Aebersold R (2003). A statistical model for identifying proteins by tandem mass spectrometry. Anal Chem.

[47] Han DK, Eng J, Zhou H, Aebersold R (2001). Quantitative profiling of differentiation-induced microsomal proteins using isotope-coded affinity tags and mass spectrometry. Nat Biotechnol.

[48] Cox J, Mann M (2012). 1D and 2D annotation enrichment: a statistical method integrating quantitative proteomics with complementary high-throughput data. BMC Bioinformatics.

