# Supplementary material for: Quantitative proteomic analysis of formalin–fixed, paraffin–embedded clear cell renal cell carcinoma tissue using stable isotopic dimethylation of primary amines
Source: BMC Genomics. 2015 Jul 29;16(1):559. doi: 10.1186/s12864-015-1768-x (PMC4518706; doi:10.1186/s12864-015-1768-x)
Supplement: Additional file 1: Table S1. — Proteins with significantly increased abundance in ccRCC compared to adjacent non-malignant tissue (p-value < 0.05, 2-tailed Student t-test, see main text for further details). Empty cells indicate that a protein was not identified in a replicate. Table S2. Proteins with significantly increased abundance in ccRCC compared to adjacent non-malignant tissue (p-value < 0.05, 2-tailed Student t-test, see main text for further details). Empty cells indicate that a protein was not identified in a replicate. [file 12864_2015_1768_MOESM1_ESM.docx]

**Weißer et al, Sup. Table 1**

Proteins with significantly increased abundance in ccRCC compared to adjacent non-malignant tissue (p-value < 0.05, 2-tailed Student t-test, see main text for further details). Empty cells indicate that a protein was not identified in a replicate.

| **Uniprot ID** | **Name** | **Fc-value** | **p-value** | **Replicate 1** | **Replicate 2** | **Replicate 3** | **Replicate 4** |
| --- | --- | --- | --- | --- | --- | --- | --- |
| CLIC1_HUMAN | Chloride intracellular channel protein 1 | 0.95 | 0.01 | 1.10 | 1.00 | 0.55 | 1.15 |
| EIF3F_HUMAN | Eukaryotic translation initiation factor 3 subunit F {ECO:0000255\|HAMAP-Rule:MF_03005} | 0.84 | 0.05 | 0.63 | 0.31 | 1.51 | 0.93 |
| IMPA2_HUMAN | Inositol monophosphatase 2 | 0.95 | 0.05 | 0.94 |  | 1.33 | 0.57 |
| PSA7_HUMAN | Proteasome subunit alpha type-7 | 0.94 | 0.01 | 1.02 | 0.44 | 1.10 | 1.19 |
| TPSN_HUMAN | Tapasin | 2.08 | 0.03 | 1.73 |  | 1.76 | 2.76 |
| TPD54_HUMAN | Tumor protein D54 | 1.14 | 0.01 | 1.36 | 1.11 | 1.55 | 0.56 |
| H2B1K_HUMAN | Histone H2B type 1-K | 1.31 | 0.05 | 0.31 | 2.30 | 1.40 | 1.24 |
| PDCD6_HUMAN | Programmed cell death protein 6 | 1.12 | 0.01 |  | 1.04 | 1.35 | 0.97 |
| PRAF3_HUMAN | PRA1 family protein 3 | 0.99 | 0.03 | 1.61 | 1.00 | 1.02 | 0.35 |
| CDS2_HUMAN | Phosphatidate cytidylyltransferase 2 | 1.13 | 0.00 |  | 1.26 | 1.11 | 1.03 |
| LDHA_HUMAN | L-lactate dehydrogenase A chain | 1.97 | 0.04 | 2.83 | 0.54 | 2.77 | 1.76 |
| CRYAB_HUMAN | Alpha-crystallin B chain | 1.70 | 0.05 | 1.61 | 2.00 | 2.85 | 0.35 |
| ALDOA_HUMAN | Fructose-bisphosphate aldolase A | 1.71 | 0.00 | 2.28 | 1.64 | 1.44 | 1.48 |
| RLA1_HUMAN | 60S acidic ribosomal protein P1 | 1.10 | 0.02 |  | 1.31 | 0.76 | 1.23 |
| RLA2_HUMAN | 60S acidic ribosomal protein P2 | 1.32 | 0.03 | 2.29 | 0.84 | 0.76 | 1.41 |
| RLA0_HUMAN | 60S acidic ribosomal protein P0 | 0.89 | 0.01 | 1.09 | 1.26 | 0.59 | 0.63 |
| NPM_HUMAN | Nucleophosmin | 1.10 | 0.02 | 0.64 | 1.44 | 1.63 | 0.69 |
| GPX1_HUMAN | Glutathione peroxidase 1 | 0.63 | 0.03 | 0.82 | 1.00 | 0.37 | 0.35 |
| ANXA2_HUMAN | Annexin A2 | 1.44 | 0.00 | 0.93 | 1.49 | 1.70 | 1.65 |
| TBB5_HUMAN | Tubulin beta chain | 1.09 | 0.03 | 0.40 | 1.04 | 1.25 | 1.67 |
| PROF1_HUMAN | Profilin-1 | 0.95 | 0.01 | 1.06 | 0.72 | 1.35 | 0.67 |
| HS90B_HUMAN | Heat shock protein HSP 90-beta | 0.84 | 0.01 | 0.99 | 0.87 | 1.09 | 0.39 |
| RU17_HUMAN | U1 small nuclear ribonucleoprotein 70 kDa | 0.90 | 0.04 |  | 1.00 | 0.55 | 1.15 |
| RSSA_HUMAN | 40S ribosomal protein SA {ECO:0000255\|HAMAP-Rule:MF_03016} | 1.04 | 0.00 | 1.06 | 1.00 | 0.85 | 1.25 |
| ANXA4_HUMAN | Annexin A4 | 2.35 | 0.01 | 2.79 | 1.31 | 2.55 | 2.76 |
| ALDOC_HUMAN | Fructose-bisphosphate aldolase C | 1.69 | 0.01 | 2.22 | 1.35 | 2.15 | 1.05 |
| LYAG_HUMAN | Lysosomal alpha-glucosidase | 0.79 | 0.03 | 0.22 | 0.78 | 1.03 | 1.13 |
| HSP7C_HUMAN | Heat shock cognate 71 kDa protein | 0.62 | 0.04 | 0.93 | 0.90 | 0.20 | 0.43 |
| PDIA4_HUMAN | Protein disulfide-isomerase A4 | 0.87 | 0.04 | 0.82 | 0.54 | 1.60 | 0.55 |
| TCTP_HUMAN | Translationally-controlled tumor protein | 1.06 | 0.04 | 1.37 | 1.59 | 1.14 | 0.16 |
| MIF_HUMAN | Macrophage migration inhibitory factor | 1.48 | 0.00 | 1.14 | 1.49 | 1.80 | 1.50 |
| KPYM_HUMAN | Pyruvate kinase PKM | 2.01 | 0.01 | 2.95 | 1.04 | 1.94 | 2.12 |
| STMN1_HUMAN | Stathmin | 1.13 | 0.01 | 0.50 | 1.35 | 1.16 | 1.52 |
| LEG3_HUMAN | Galectin-3 | 0.72 | 0.00 | 0.69 | 0.61 | 0.90 | 0.68 |
| RL35A_HUMAN | 60S ribosomal protein L35a | 1.14 | 0.02 | 1.67 | 0.87 | 0.53 | 1.48 |
| PGAM1_HUMAN | Phosphoglycerate mutase 1 | 0.81 | 0.03 | 0.72 | 1.40 | 0.74 | 0.38 |
| PSB1_HUMAN | Proteasome subunit beta type-1 | 0.96 | 0.02 | 0.59 | 0.87 | 0.85 | 1.55 |
| PPIB_HUMAN | Peptidyl-prolyl cis-trans isomerase B | 0.74 | 0.02 | 1.17 | 0.75 | 0.69 | 0.36 |
| RS12_HUMAN | 40S ribosomal protein S12 | 0.99 | 0.04 | 0.61 | 1.23 |  | 1.15 |
| PSA2_HUMAN | Proteasome subunit alpha type-2 | 0.70 | 0.03 | 1.19 | 0.35 | 0.57 | 0.68 |
| PSA4_HUMAN | Proteasome subunit alpha type-4 | 0.76 | 0.04 |  | 0.44 | 0.88 | 0.95 |
| SYVC_HUMAN | Valine--tRNA ligase | 0.79 | 0.01 | 1.09 | 0.84 | 0.69 | 0.54 |
| PSB8_HUMAN | Proteasome subunit beta type-8 | 1.29 | 0.04 | 1.48 | 0.25 | 1.47 | 1.94 |
| PSB9_HUMAN | Proteasome subunit beta type-9 | 2.07 | 0.04 | 2.42 | 0.49 | 3.18 | 2.20 |
| PSA5_HUMAN | Proteasome subunit alpha type-5 | 0.83 | 0.00 | 0.86 | 0.51 | 0.98 | 0.95 |
| MARCS_HUMAN | Myristoylated alanine-rich C-kinase substrate | 1.90 | 0.03 | 1.92 | 0.54 | 2.75 | 2.39 |
| RL9_HUMAN | 60S ribosomal protein L9 | 0.86 | 0.02 | 1.32 | 0.72 | 0.90 | 0.50 |
| RL22_HUMAN | 60S ribosomal protein L22 | 1.23 | 0.00 | 1.46 | 0.81 | 1.40 | 1.27 |
| RL4_HUMAN | 60S ribosomal protein L4 | 0.78 | 0.04 | 1.19 | 1.04 | 0.20 | 0.68 |
| RS19_HUMAN | 40S ribosomal protein S19 | 0.86 | 0.02 | 1.44 | 0.75 | 0.48 | 0.79 |
| OST48_HUMAN | Dolichyl-diphosphooligosaccharide--protein glycosyltransferase 48 kDa subunit | 0.78 | 0.02 | 1.20 | 0.37 | 0.59 | 0.94 |
| RS9_HUMAN | 40S ribosomal protein S9 | 0.89 | 0.03 | 0.76 | 0.49 | 1.57 | 0.74 |
| RS5_HUMAN | 40S ribosomal protein S5 | 1.08 | 0.04 | 1.20 | 1.26 | 0.17 | 1.67 |
| TCPE_HUMAN | T-complex protein 1 subunit epsilon | 0.65 | 0.03 | 0.92 | 0.25 | 0.91 | 0.55 |
| SYSC_HUMAN | Serine--tRNA ligase, cytoplasmic | 0.66 | 0.03 | 0.86 | 0.59 | 0.24 | 0.94 |
| PSB3_HUMAN | Proteasome subunit beta type-3 | 0.91 | 0.02 | 1.46 | 0.61 | 0.98 | 0.61 |
| PSB2_HUMAN | Proteasome subunit beta type-2 | 0.97 | 0.03 | 1.28 | 0.49 | 0.61 | 1.50 |
| BIEA_HUMAN | Biliverdin reductase A | 1.16 | 0.02 | 1.03 | 0.78 | 0.98 | 1.86 |
| TCP4_HUMAN | Activated RNA polymerase II transcriptional coactivator p15 | 1.01 | 0.03 | 1.49 | 0.33 | 1.41 | 0.84 |
| MANF_HUMAN | Mesencephalic astrocyte-derived neurotrophic factor | 1.37 | 0.01 | 1.14 | 1.59 |  | 1.38 |
| SUMO3_HUMAN | Small ubiquitin-related modifier 3 {ECO:0000305} | 1.03 | 0.04 | 0.63 | 1.07 |  | 1.39 |
| IF6_HUMAN | Eukaryotic translation initiation factor 6 {ECO:0000255\|HAMAP-Rule:MF_03132} | 0.79 | 0.00 | 0.82 | 0.64 | 0.71 | 1.02 |
| IF4A1_HUMAN | Eukaryotic initiation factor 4A-I | 0.87 | 0.01 | 0.59 | 1.15 | 0.80 | 0.96 |
| UBC12_HUMAN | NEDD8-conjugating enzyme Ubc12 | 0.77 | 0.02 | 0.89 | 0.54 |  | 0.88 |
| ARP3_HUMAN | Actin-related protein 3 | 0.71 | 0.04 | 0.92 | 0.07 | 0.97 | 0.89 |
| RL15_HUMAN | 60S ribosomal protein L15 | 0.77 | 0.00 | 0.73 | 0.51 | 0.97 | 0.86 |
| UFM1_HUMAN | Ubiquitin-fold modifier 1 | 0.96 | 0.03 | 0.96 | 0.31 | 1.31 | 1.28 |
| NTF2_HUMAN | Nuclear transport factor 2 | 1.02 | 0.01 | 0.88 | 0.90 | 0.77 | 1.53 |
| 1433G_HUMAN | 14-3-3 protein gamma | 0.73 | 0.00 | 0.94 | 0.54 | 0.61 | 0.83 |
| RS8_HUMAN | 40S ribosomal protein S8 | 0.88 | 0.01 | 1.22 | 0.90 | 0.48 | 0.92 |
| RS16_HUMAN | 40S ribosomal protein S16 | 0.99 | 0.03 | 1.58 | 0.37 | 0.91 | 1.09 |
| RS14_HUMAN | 40S ribosomal protein S14 | 0.94 | 0.03 | 1.42 | 0.81 | 0.31 | 1.23 |
| RS13_HUMAN | 40S ribosomal protein S13 | 0.81 | 0.05 | 1.25 | 0.97 | 0.10 | 0.92 |
| RS11_HUMAN | 40S ribosomal protein S11 | 0.76 | 0.02 | 1.18 | 0.46 | 0.46 | 0.93 |
| RUXE_HUMAN | Small nuclear ribonucleoprotein E | 1.32 | 0.04 |  | 1.81 | 0.94 | 1.20 |
| LSM3_HUMAN | U6 snRNA-associated Sm-like protein LSm3 | 1.07 | 0.03 | 0.61 | 1.04 | 0.80 | 1.84 |
| SMD2_HUMAN | Small nuclear ribonucleoprotein Sm D2 | 0.86 | 0.03 | 0.55 | 0.90 | 0.51 | 1.48 |
| RL23A_HUMAN | 60S ribosomal protein L23a | 0.91 | 0.02 | 0.71 | 0.87 |  | 1.14 |
| RS6_HUMAN | 40S ribosomal protein S6 | 0.85 | 0.00 | 0.83 | 0.64 | 1.03 | 0.88 |
| RAN_HUMAN | GTP-binding nuclear protein Ran | 0.61 | 0.03 | 0.31 | 0.78 | 0.37 | 0.99 |
| RL23_HUMAN | 60S ribosomal protein L23 | 1.09 | 0.03 | 1.78 | 0.93 | 0.51 | 1.13 |
| RS25_HUMAN | 40S ribosomal protein S25 | 0.95 | 0.02 | 1.16 | 0.75 |  | 0.93 |
| RS30_HUMAN | 40S ribosomal protein S30 | 1.16 | 0.02 | 1.82 | 0.72 | 1.12 | 0.96 |
| RL31_HUMAN | 60S ribosomal protein L31 | 1.07 | 0.02 | 1.71 | 0.75 | 0.59 | 1.24 |
| RL11_HUMAN | 60S ribosomal protein L11 | 0.86 | 0.04 | 1.49 | 0.39 | 0.53 | 1.04 |
| RL8_HUMAN | 60S ribosomal protein L8 | 1.07 | 0.03 | 1.82 | 0.67 | 0.64 | 1.14 |
| 1433Z_HUMAN | 14-3-3 protein zeta/delta | 0.68 | 0.00 | 0.68 | 0.69 | 0.90 | 0.47 |
| GBLP_HUMAN | Guanine nucleotide-binding protein subunit beta-2-like 1 | 0.90 | 0.03 | 1.35 | 1.07 | 0.27 | 0.93 |
| H31_HUMAN | Histone H3.1 | 1.14 | 0.01 | 0.80 | 0.75 | 1.56 | 1.44 |
| RL24_HUMAN | 60S ribosomal protein L24 | 0.84 | 0.05 | 1.22 | 0.64 |  | 0.65 |
| RL19_HUMAN | 60S ribosomal protein L19 | 1.22 | 0.03 | 1.23 | 0.69 | 2.08 | 0.90 |
| K6PP_HUMAN | ATP-dependent 6-phosphofructokinase, platelet type {ECO:0000255\|HAMAP-Rule:MF_03184} | 2.39 | 0.02 | 1.36 | 1.69 | 2.91 | 3.61 |
| DSC2_HUMAN | Desmocollin-2 | 2.10 | 0.01 |  | 2.30 | 1.68 | 2.31 |
| 1433F_HUMAN | 14-3-3 protein eta | 1.08 | 0.05 | 0.10 | 1.40 | 1.53 | 1.29 |
| RL18_HUMAN | 60S ribosomal protein L18 | 0.87 | 0.01 | 1.12 | 1.07 | 0.51 | 0.77 |
| CBX3_HUMAN | Chromobox protein homolog 3 | 0.87 | 0.02 |  | 1.04 | 0.98 | 0.60 |
| NACA_HUMAN | Nascent polypeptide-associated complex subunit alpha | 0.80 | 0.00 | 0.51 | 0.84 | 0.85 | 1.00 |
| MVP_HUMAN | Major vault protein | 1.60 | 0.00 | 1.29 | 1.40 | 1.77 | 1.96 |
| ELAV1_HUMAN | ELAV-like protein 1 | 0.68 | 0.05 | 0.06 | 0.81 | 0.79 | 1.04 |
| TRXR1_HUMAN | Thioredoxin reductase 1, cytoplasmic | 1.30 | 0.02 | 1.60 | 0.67 | 1.86 | 1.08 |
| SND1_HUMAN | Staphylococcal nuclease domain-containing protein 1 | 0.86 | 0.05 | 1.00 | 0.46 |  | 1.11 |
| THOC4_HUMAN | THO complex subunit 4 | 0.93 | 0.03 |  | 1.11 | 0.62 | 1.07 |
| DLRB2_HUMAN | Dynein light chain roadblock-type 2 | 0.76 | 0.01 | 0.90 | 0.61 | 0.44 | 1.11 |
| GSLG1_HUMAN | Golgi apparatus protein 1 | 1.09 | 0.00 | 1.05 | 1.35 | 0.69 | 1.25 |
| KCD12_HUMAN | BTB/POZ domain-containing protein KCTD12 | 0.94 | 0.02 | 0.35 | 1.35 | 1.09 | 0.95 |
| PSMD1_HUMAN | 26S proteasome non-ATPase regulatory subunit 1 | 0.63 | 0.02 | 0.51 | 0.59 |  | 0.79 |
| PLIN2_HUMAN | Perilipin-2 | 2.82 | 0.02 | 2.78 | 1.00 | 4.13 | 3.37 |
| APOL2_HUMAN | Apolipoprotein L2 | 1.85 | 0.01 | 2.11 | 1.64 | 1.79 |  |
| TMED9_HUMAN | Transmembrane emp24 domain-containing protein 9 | 1.39 | 0.02 | 1.55 | 0.67 | 2.06 | 1.28 |
| RTN4_HUMAN | Reticulon-4 | 0.99 | 0.00 | 0.68 | 1.00 | 1.09 | 1.20 |
| PSME2_HUMAN | Proteasome activator complex subunit 2 | 0.63 | 0.03 | 0.79 | 0.13 | 0.72 | 0.90 |
| RL36_HUMAN | 60S ribosomal protein L36 | 1.44 | 0.02 | 1.72 | 1.54 |  | 1.07 |

**Weißer et al, Sup. Table 2**

Proteins with significantly increased abundance in ccRCC compared to adjacent non-malignant tissue (p-value < 0.05, 2-tailed Student t-test, see main text for further details). Empty cells indicate that a protein was not identified in a replicate.

| **Uniprot** | **Name** | **Fc-value** | **p-value** | **Replicate 1** | **Replicate 2** | **Replicate 3** | **Replicate 4** |
| --- | --- | --- | --- | --- | --- | --- | --- |
| MGA_HUMAN | Maltase-glucoamylase, intestinal | -1.79 | 0.01 |  | -1.68 | -1.64 | -2.05 |
| CS077_HUMAN | Small integral membrane protein 24 | -2.80 | 0.04 |  | -3.97 | -2.15 | -2.29 |
| CAD16_HUMAN | Cadherin-16 | -1.83 | 0.00 | -1.68 | -1.86 | -1.83 | -1.94 |
| ENTP5_HUMAN | Ectonucleoside triphosphate diphosphohydrolase 5 | -2.15 | 0.02 |  | -1.86 | -2.73 | -1.84 |
| AL1L1_HUMAN | Cytosolic 10-formyltetrahydrofolate dehydrogenase | -1.53 | 0.02 |  | -1.87 | -1.21 | -1.50 |
| BODG_HUMAN | Gamma-butyrobetaine dioxygenase | -2.26 | 0.02 | -3.20 | -2.87 | -1.41 | -1.56 |
| FTCD_HUMAN | Formimidoyltransferase-cyclodeaminase | -1.91 | 0.02 |  | -2.39 | -1.64 | -1.70 |
| NAPSA_HUMAN | Napsin-A | -1.21 | 0.04 |  | -1.55 | -0.69 | -1.38 |
| PLMN_HUMAN | Plasminogen | -0.82 | 0.02 | -1.27 | -0.99 | -0.41 | -0.62 |
| ASSY_HUMAN | Argininosuccinate synthase | -3.13 | 0.00 |  | -2.77 | -3.15 | -3.48 |
| A1AT_HUMAN | Alpha-1-antitrypsin | -1.02 | 0.01 | -0.73 | -1.06 | -0.88 | -1.40 |
| HV303_HUMAN | Ig heavy chain V-III region VH26 | -1.59 | 0.00 | -1.68 | -1.62 | -1.49 |  |
| APOE_HUMAN | Apolipoprotein E | -0.90 | 0.03 | -1.45 | -0.95 | -0.27 | -0.91 |
| AMBP_HUMAN | Protein AMBP | -1.28 | 0.03 | -0.83 | -2.08 | -0.69 | -1.50 |
| FETUA_HUMAN | Alpha-2-HS-glycoprotein | -0.76 | 0.03 | -0.28 | -0.80 | -1.21 | -0.74 |
| MT2_HUMAN | Metallothionein-2 | -2.95 | 0.05 | -0.56 | -2.94 | -3.41 | -4.90 |
| VTNC_HUMAN | Vitronectin | -1.33 | 0.01 | -1.65 | -1.89 | -0.83 | -0.96 |
| AT1B1_HUMAN | Sodium/potassium-transporting ATPase subunit beta-1 | -1.42 | 0.03 | -0.57 | -1.16 | -1.74 | -2.19 |
| ALDOB_HUMAN | Fructose-bisphosphate aldolase B | -4.26 | 0.01 | -2.67 | -6.08 | -3.15 | -5.15 |
| PCCA_HUMAN | Propionyl-CoA carboxylase alpha chain, mitochondrial | -1.58 | 0.05 |  | -0.89 | -1.83 | -2.03 |
| PCCB_HUMAN | Propionyl-CoA carboxylase beta chain, mitochondrial | -1.32 | 0.05 | -0.57 | -0.94 | -1.34 | -2.45 |
| CALB1_HUMAN | Calbindin | -3.14 | 0.01 |  | -3.09 | -2.73 | -3.59 |
| FABPL_HUMAN | Fatty acid-binding protein, liver | -4.60 | 0.03 |  | -5.61 | -2.93 | -5.24 |
| NEP_HUMAN | Neprilysin | -2.85 | 0.02 |  | -3.76 | -2.42 | -2.37 |
| ADT1_HUMAN | ADP/ATP translocase 1 | -1.57 | 0.00 | -1.65 |  | -1.41 | -1.66 |
| NQO2_HUMAN | Ribosyldihydronicotinamide dehydrogenase [quinone] | -1.02 | 0.00 |  | -0.94 | -1.15 | -0.99 |
| ACADS_HUMAN | Short-chain specific acyl-CoA dehydrogenase, mitochondrial | -1.68 | 0.01 |  | -1.60 | -2.04 | -1.39 |
| DPEP1_HUMAN | Dipeptidase 1 | -1.87 | 0.04 |  | -1.07 | -2.15 | -2.39 |
| DDC_HUMAN | Aromatic-L-amino-acid decarboxylase | -2.48 | 0.01 |  | -2.74 | -2.04 | -2.67 |
| GSTM3_HUMAN | Glutathione S-transferase Mu 3 | -1.86 | 0.03 | -2.67 | -1.17 | -0.88 | -2.74 |
| AOFA_HUMAN | Amine oxidase [flavin-containing] A | -1.37 | 0.03 | -1.91 | -1.07 | -0.53 | -1.95 |
| GPDA_HUMAN | Glycerol-3-phosphate dehydrogenase [NAD(+)], cytoplasmic | -2.40 | 0.00 | -2.45 | -2.48 | -1.56 | -3.10 |
| ZA2G_HUMAN | Zinc-alpha-2-glycoprotein | -0.75 | 0.00 | -0.62 | -1.01 | -0.65 | -0.73 |
| AOFB_HUMAN | Amine oxidase [flavin-containing] B | -0.95 | 0.03 |  | -0.82 | -1.28 | -0.76 |
| DPP4_HUMAN | Dipeptidyl peptidase 4 | -0.94 | 0.03 | -1.45 | -0.49 | -0.60 | -1.21 |
| PSB5_HUMAN | Proteasome subunit beta type-5 | -0.76 | 0.01 | -0.74 | -0.44 | -1.03 | -0.81 |
| GNA11_HUMAN | Guanine nucleotide-binding protein subunit alpha-11 | -1.08 | 0.03 | -1.45 | -0.79 |  | -1.01 |
| AL4A1_HUMAN | Delta-1-pyrroline-5-carboxylate dehydrogenase, mitochondrial | -2.36 | 0.01 | -1.62 | -1.87 | -2.56 | -3.39 |
| PBLD_HUMAN | Phenazine biosynthesis-like domain-containing protein | -2.10 | 0.03 |  | -2.78 | -1.74 | -1.79 |
| GLPK_HUMAN | Glycerol kinase | -1.49 | 0.04 |  | -2.05 | -1.03 | -1.39 |
| GLRX1_HUMAN | Glutaredoxin-1 | -0.84 | 0.03 |  | -0.95 | -0.56 | -1.02 |
| THIM_HUMAN | 3-ketoacyl-CoA thiolase, mitochondrial | -1.23 | 0.01 | -1.09 | -0.84 | -1.41 | -1.59 |
| ACDSB_HUMAN | Short/branched chain specific acyl-CoA dehydrogenase, mitochondrial | -1.41 | 0.01 |  | -1.66 | -1.49 | -1.07 |
| IDHP_HUMAN | Isocitrate dehydrogenase [NADP], mitochondrial | -1.31 | 0.01 | -0.84 | -0.95 | -1.64 | -1.81 |
| ACOT2_HUMAN | Acyl-coenzyme A thioesterase 2, mitochondrial | -1.40 | 0.03 |  | -1.78 | -0.93 | -1.48 |
| KHK_HUMAN | Ketohexokinase | -1.88 | 0.02 |  | -2.09 | -2.15 | -1.41 |
| SSDH_HUMAN | Succinate-semialdehyde dehydrogenase, mitochondrial | -0.76 | 0.05 | -0.27 | -0.94 | -0.49 | -1.33 |
| LYSC_HUMAN | Lysozyme C | -1.38 | 0.01 | -0.65 | -1.93 | -1.56 | -1.37 |
| GABT_HUMAN | 4-aminobutyrate aminotransferase, mitochondrial | -2.22 | 0.04 |  | -1.29 | -2.56 | -2.81 |
| DAB2_HUMAN | Disabled homolog 2 | -1.00 | 0.03 |  | -1.36 | -0.88 | -0.76 |
| ODO1_HUMAN | 2-oxoglutarate dehydrogenase, mitochondrial | -1.25 | 0.04 | -1.40 | -0.24 | -1.41 | -1.97 |
| MMSA_HUMAN | Methylmalonate-semialdehyde dehydrogenase [acylating], mitochondrial | -2.03 | 0.03 | -1.25 | -1.43 | -2.04 | -3.42 |
| ACY1_HUMAN | Aminoacylase-1 | -2.00 | 0.03 | -1.09 | -1.64 | -1.92 | -3.34 |
| ACS2A_HUMAN | Acyl-coenzyme A synthetase ACSM2A, mitochondrial | -3.05 | 0.01 | -2.81 | -1.84 | -2.93 | -4.62 |
| AUHM_HUMAN | Methylglutaconyl-CoA hydratase, mitochondrial | -1.73 | 0.04 |  | -2.26 | -1.83 | -1.11 |
| DPYS_HUMAN | Dihydropyrimidinase | -1.43 | 0.02 |  | -1.73 | -1.09 | -1.48 |
| PCKGM_HUMAN | Phosphoenolpyruvate carboxykinase [GTP], mitochondrial | -2.39 | 0.04 | -0.76 | -2.18 | -2.42 | -4.19 |
| HCDH_HUMAN | Hydroxyacyl-coenzyme A dehydrogenase, mitochondrial | -1.47 | 0.01 | -1.25 | -1.16 | -1.15 | -2.34 |
| ACS2B_HUMAN | Acyl-coenzyme A synthetase ACSM2B, mitochondrial | -2.06 | 0.01 |  | -2.38 | -1.64 | -2.15 |
| HIBCH_HUMAN | 3-hydroxyisobutyryl-CoA hydrolase, mitochondrial | -1.37 | 0.04 | -0.54 | -0.98 | -1.74 | -2.21 |
| S22A8_HUMAN | Solute carrier family 22 member 8 | -1.99 | 0.02 |  | -2.35 | -2.15 | -1.47 |
| GLYL1_HUMAN | Glycine N-acyltransferase-like protein 1 | -3.29 | 0.03 |  | -4.20 | -3.41 | -2.27 |
| ISOC2_HUMAN | Isochorismatase domain-containing protein 2, mitochondrial | -1.25 | 0.04 |  | -0.94 | -1.74 | -1.08 |
| GALM_HUMAN | Aldose 1-epimerase | -1.16 | 0.01 |  | -0.96 | -1.15 | -1.37 |
| ACSF2_HUMAN | Acyl-CoA synthetase family member 2, mitochondrial | -3.08 | 0.04 |  | -3.90 | -1.92 | -3.42 |
| PDLI2_HUMAN | PDZ and LIM domain protein 2 | -1.94 | 0.05 |  | -2.62 | -1.09 | -2.11 |
| HCD2_HUMAN | 3-hydroxyacyl-CoA dehydrogenase type-2 | -1.10 | 0.01 | -0.77 | -0.87 | -1.28 | -1.49 |
| SPEB_HUMAN | Agmatinase, mitochondrial | -2.68 | 0.01 |  | -2.95 | -2.93 | -2.16 |
| HDHD3_HUMAN | Haloacid dehalogenase-like hydrolase domain-containing protein 3 | -2.30 | 0.02 |  | -1.73 | -2.56 | -2.61 |
| BDH2_HUMAN | 3-hydroxybutyrate dehydrogenase type 2 | -1.19 | 0.00 | -1.42 | -0.91 | -1.15 | -1.27 |
| HAOX2_HUMAN | Hydroxyacid oxidase 2 | -2.50 | 0.02 |  | -3.09 | -1.74 | -2.67 |
| SUCB1_HUMAN | Succinyl-CoA ligase [ADP-forming] subunit beta, mitochondrial | -0.88 | 0.02 | -1.34 | -0.56 | -0.53 | -1.07 |
| GBG12_HUMAN | Guanine nucleotide-binding protein G(I)/G(S)/G(O) subunit gamma-12 | -0.65 | 0.04 | -1.20 | -0.41 | -0.38 | -0.63 |
| PROD2_HUMAN | Probable proline dehydrogenase 2 | -1.99 | 0.00 |  | -1.78 | -2.15 | -2.03 |
| NAT8_HUMAN | N-acetyltransferase 8 | -2.69 | 0.03 |  | -2.85 | -1.74 | -3.48 |
| S23A1_HUMAN | Solute carrier family 23 member 1 | -3.01 | 0.05 |  | -3.31 | -1.74 | -3.98 |
| VATH_HUMAN | V-type proton ATPase subunit H | -1.19 | 0.00 | -1.01 | -0.92 | -1.34 | -1.47 |
